# Effect of Phenotypic Screening of Extracts and Fractions of* Erythrophleum ivorense* Leaf and Stem Bark on Immature and Adult Stages of* Schistosoma mansoni*

**DOI:** 10.1155/2018/9431467

**Published:** 2018-06-07

**Authors:** Gertrude Kyere-Davies, Christian Agyare, Yaw Duah Boakye, Brian M. Suzuki, Conor R. Caffrey

**Affiliations:** ^1^Department of Pharmaceutics, Faculty of Pharmacy and Pharmaceutical Sciences, Kwame Nkrumah University of Science and Technology, Kumasi, Ghana; ^2^Center for Discovery and Innovation in Parasitic Diseases (CDIPD), Skaggs School of Pharmacy and Pharmaceutical Sciences, University of California San Diego, La Jolla, CA 92093, USA

## Abstract

Schistosomiasis is a disease caused by a flatworm parasite that infects people in tropical and subtropical regions of Sub-Saharan Africa, South America, China, and Southeast Asia. The reliance on just one drug for current treatment emphasizes the need for new chemotherapeutic strategies. The aim of this study was to determine the phenotypic effects of extracts and fractions of leaf and stem bark of* Erythrophleum ivorense *(family Euphorbiaceae), a tree that grows in tropical parts of Africa, on two developmental stages of* Schistosoma mansoni*, namely, postinfective larvae (schistosomula or somules) and adults. Methanol leaf and stem bark extracts of* E. ivorense *were successively fractionated with acetone, petroleum ether, ethyl acetate, and methanol. These fractions were then incubated with somules at 0.3125 to 100 *μ*g/mL and with adults at 1.25 *μ*g/mL. The acetone fractions of both the methanol leaf and bark of* E. ivorense *were most active against the somules whereas the petroleum ether fractions showed least activity. For adult parasites, the acetone fraction of methanol bark extract also elicited phenotypic changes. The data arising provide the first step in the discovery of new treatments for an endemic infectious disease using locally sourced African medicinal plants.

## 1. Introduction

Schistosomiasis, also known as bilharzia, is a disease caused by blood flukes of the genus* Schistosoma *[1, 2]. There are five species of* Schistosoma* that infect humans:* Schistosoma mansoni*,* Schistosoma haematobium, Schistosoma japonicum, Schistosoma intercalatum, *and* Schistosoma mekongi *with the first three being the most common [3, 4]. The disease is chronic and morbid [5] and affects people in tropical and subtropical regions of Sub-Saharan Africa, South America, China, and Southeast Asia [6, 7]. It is estimated that about 200 million people are infected with another 400 million being at risk [8]. Schistosomiasis is a persistent public health issue in endemic countries.

In Ghana, schistosomiasis is caused mainly by* S. haematobium* and* S. mansoni* [9] and is known to affect mostly school-aged children from 10 years of age, particularly, boys. High prevalence of the disease (11 and 64%) has been reported in the Kumasi metropolitan area of Ghana [10]. Aryeetey et al. [11] also reported prevalence rates of 54.8 to 60.0% for urinary schistosomiasis (caused by* S. haematobium*) in the Akuapim South district of Eastern Ghana. Nkegbe [12] reported that about 50% of the people in the Volta and Greater Accra regions of Ghana suffer from schistosomiasis. Approximately 48% of school-aged children are infected with schistosomiasis in the rural north of Ghana [13].

Currently, the treatment of schistosomiasis relies upon just one drug, praziquantel, which is safe and reasonably effective [6, 7]. However, side effects, including headache, dizziness, stomach, joint and muscle pain, tiredness, weakness, skin rashes, and sweating, are common [7]. Also, the reliance on a single antimicrobial agent could precipitate resistance. It is, therefore, important to discover and develop new chemical molecules that could be used in place of or together with praziquantel.

*Erythrophleum ivorense* A. Chev. is a tree that grows in tropical parts of Africa, including Ghana, Congo, Cameroon, Gabon, Nigeria, and Liberia. It is a legume from the Euphorbiaceae family [14]. Phytochemical screening of methanol extracts of both leaves and bark reveals the presence of saponins and flavonoids. The methanol leaf extract contains condensed tannins and sterols whereas the methanol bark extract contains hydrolysable tannins and terpenoids [14].* E. ivorense *contains alkaloids including erythrophleine [15]. Medicinally, it is used as an emetic, an analgesic, and a laxative in Sierra Leone [16]. It also possesses broad-spectrum antimicrobial activity [14]. The aim of this study was to phenotypically screen* E. ivorense *leaf and stem bark extracts and fractions against various developmental stages of* S. mansoni.*

## 2. Methods

### 2.1. Collection of Plant Material

The leaves and barks of* E. ivorense *were collected from the Botanic Garden, University of Ghana, in January 2015 by Mr. John Yaw Amponsah and authenticated by Professor Alex Asase of the Department of Botany of the University of Ghana, Legon. A voucher specimen (AA 45) of the plant material was kept in the Ghana Herbarium, University of Ghana, Legon, Accra, Ghana.

### 2.2. Preparation of Extracts and Fractions

The leaf and stem bark of* E. ivorense *were extracted using 99.8% v/v methanol (Sigma-Aldrich, MO, USA). Acetone (96% v/v), petroleum ether (96 v/v), ethyl acetate (98% v/v), and methanol (all solvents purchased from Sigma-Aldrich, MO, USA) were used for the successive extraction and fractionation of the methanol leaf and bark extracts using column chromatography. The extracts were prepared by the cold maceration of 300 g of powdered dry plant material in stoppered flasks containing 700 mL of solvent for 1 week at room temperature (28°C). After filtration, the solvent was evaporated under reduced pressure in a rotary evaporator at 40° C. The different extracts and fractions were conserved in tightly sealed glass vials and stored at 4°C.

### 2.3. Maintenance of* S. Mansoni*

A Puerto Rican isolate of* Schistosoma mansoni* was maintained by passage through* Biomphalaria glabrata* snails and 3-5-week-old female Golden Syrian hamsters as intermediate and definitive hosts, respectively [2, 17]. Infected snails were induced with light to shed infectious larvae (cercariae), as described by Colley and Wikel [18], and the cercariae were mechanically transformed to postinfective larvae (schistosomula or somules), as described Stefanic et al. [19]. Adults were harvested from euthanized hamsters at 6 weeks postinfection by reverse perfusion in RPMI 1640 medium [20].

### 2.4. *Schistosoma mansoni *Phenotypic Screening

For screening of somules and adults, transparent u-bottomed 96-well plates and flat-bottomed 24-well plates were used, respectively. The phenotypic screening was carried out as described [2, 17].

For first-pass screens with somules, extracts, and fractions were spotted into the wells to yield a final concentration of 100 *μ*g/mL (1 *μ*L of 20 mg/mL stock solution). The final concentration of dimethyl sulfoxide (DMSO; Sigma-Aldrich, MO, USA) was less than 0.5 % v/v. Somules were then added at a density of 30 to 40 parasites in 200 *μ*L of Basch medium [21] supplemented with 2.5% v/v FBS, 100 U/mL penicillin, and 100 mg/mL streptomycin (Gibco, Carlsbad, USA) [2]. Parasites were incubated in a humidified atmosphere of 5% CO_2_ at 37°C for 72 h. For dose-response tests, extracts, and fractions that elicited a phenotypic response were screened between 0.3125 and 100 *μ*g/mL. Both first-pass and confirmatory dose-response assays were performed in duplicate. For screens with adult parasites, four to five worm pairs were incubated in the presence of 1.25 *μ*g/mL extracts or fractions (final DMSO 0.5% v/v) in 2 mL complete Basch medium.

Phenotypic changes were visually recorded at 24 and 48 h for somules and at 3, 5, 24, and 48 h with adults, as described by Glaser et al. [22] and Fonseca et al. [23] using a Zeiss Axiovert A1 inverted microscope (10X magnification for the somules and 2.5X magnification for the adults; Carl Zeiss Microscopy, Thornwood, USA). As complex metazoans, schistosomes can exhibit a variety of dynamic phenotypic responses to drug insult as a function of time and doses. Accordingly, we use single word “descriptors” to record changes in somule movement, shape, translucence, surface integrity, and, for adults specifically, the ability to remain adhered (via oral and/or ventral suckers) to the bottom of the well (Table 1). We then convert these observations into an ordinal numeric output (a “severity score”) in order to allow for the relative comparison of compound effects. Specifically, each descriptor is awarded a value of 1 up to a maximum score of 4 (Table 1). When damage to the adult parasite's tegument (surface) is evident, the maximum score of four is awarded on the understanding that such damage is lethal, including in the mammalian host [24].

## 3. Results

### 3.1. Activity of Extracts and Their Fractions of* E. ivorense* against* S. mansoni* Somules

All of the extract fractions were active at 100 *μ*g/mL and were subsequently tested in dose-response assays.

**Figure 1:**
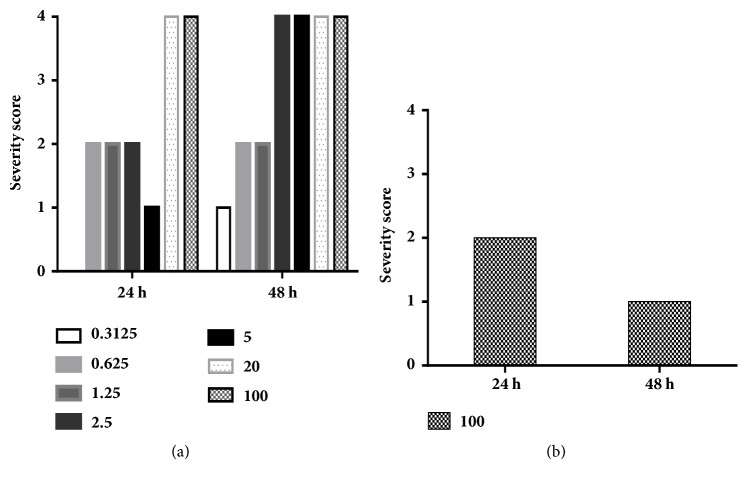
Severity scores for fractions derived from* E. ivorense* methanol leaf extracts tested against somules. Scores associated with (a) the acetone fraction and (b) the methanol extract. Note that for (b), activity was only detected at 100 *μ*g/mL.

Focusing first on the methanol leaf extract for dose-response assays, the acetone fraction was potently antischistosomal with phenotypic alterations (degeneration and/or death, i.e., severity scores of 4) noted at both 24 and 48 h and at all dilutions (except 0.3125 *μ*g/ml at 24 h; Figure 1(a); see also Figure 3(b) for a photo of the affected somules). By contrast, the methanol leaf extract elicited relative minor phenotypic alteration, only at 100 *μ*g/mL (Figure 1(b)). At 24 h, the somules were rounded and overactive yielding a score of 2 but by 48 h both effects had disappeared and the worms had become darkened, thereby yielding a score of 1. There was no apparent activity below 100 *μ*g/mL.

**Figure 2:**
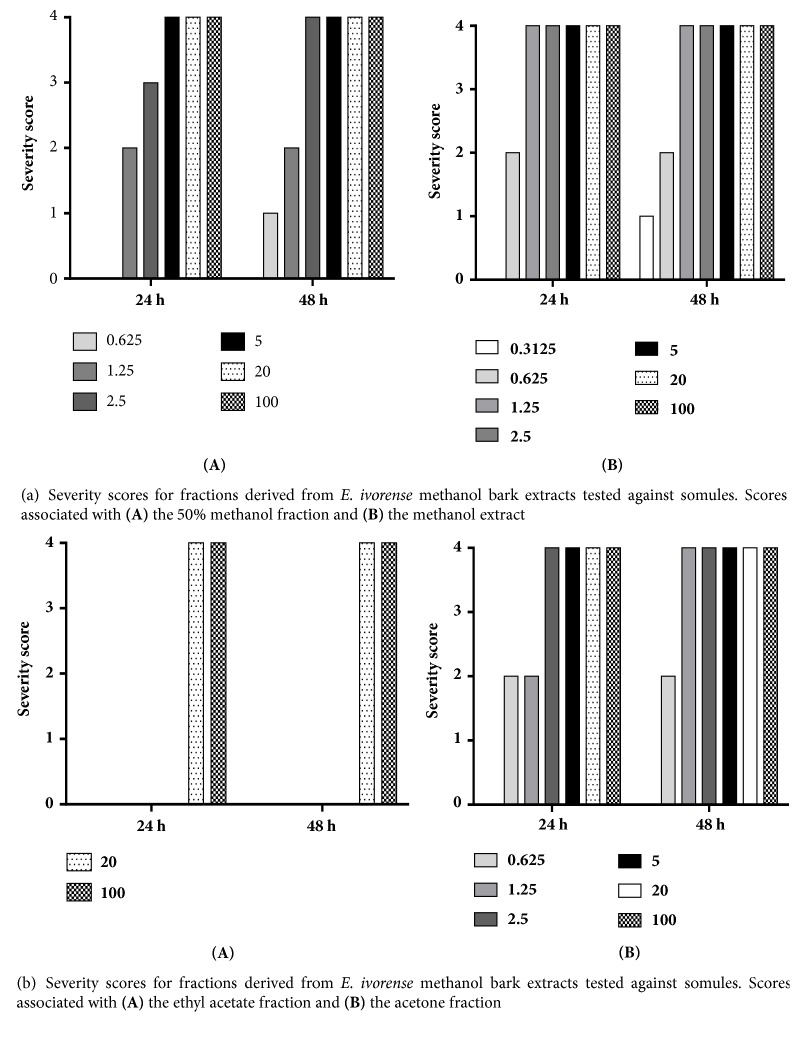


**Figure 3:**
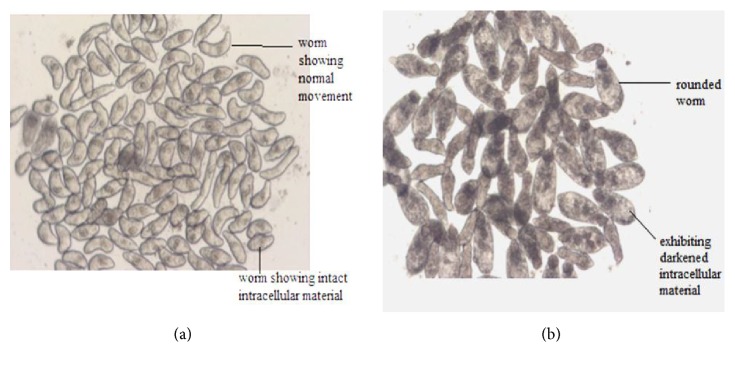
Examples of somules after exposure to E.* ivorense* extract fractions. (a) Control somules after 24 h. Each somule is approximately 200 *μ*m in length. (b) Somules exposed to 2.5 *μ*g/mL of the acetone fraction of the methanol leaf extract after 24 h. Note the rounding and severe degeneracy relative to control worms.

Focusing on the methanol stem bark extract, the 50% methanol fraction killed or caused degeneration in somules by 24 and 48 h (scores of 4) at 100, 20, 5, and 2.5 *μ*g/mL (Figure 2(a)). The least concentration that exhibited a phenotypic effect was 0.625 *μ*g/mL and then after 48 h about 50% of the worms had died giving a score of 1. No effect was observed at 24 h. At 0.3125 *μ*g/ml, the fraction was inactive. For the methanol bark extract, the somules were dead or degenerated by 24 and 48 h at 100, 20, 5, 2.5, and 1.25 *μ*g/mL (scores of 4). At a concentration of 0.625 *μ*g/mL, the worms were darkened and rounded at 24 and 48 h, respectively (severity scores of 2 for each time point). At 0.3125 *μ*g/mL after 48 h, the worms were rounded yielding a score of 1. No effect was observed after 24 h. The ethyl acetate fraction was relatively weak (Figure 2(b)). Thus, at 100 *μ*g/mL somules were killed between 24 and 48 h of incubation, yielding a severity score of 4. At 20 *μ*g/mL, the somules appeared rounded and degenerated by 24 h and were dead by 48 h (each a score of 4). Below 20 *μ*g/mL, no effect was observed. Finally, powerful effects across time and dose were noted for the acetone fraction of the methanol stem bark extract (Figure 2(b)). Specifically, concentrations of 100, 20, 5, 2.5, and 1.25 *μ*g/mL were lethal by 48 h (score of 4). At 0.625 *μ*g/mL, the somules had become rounded and darkened yielding a score of 2. At the lowest concentration tested, 0.3125 *μ*g/mL, no effects were observed (Figure 5).

### 3.2. Activity of Extracts and Their Fractions of* E. ivorense* against Adult* S. mansoni *Adults

**Figure 4:**
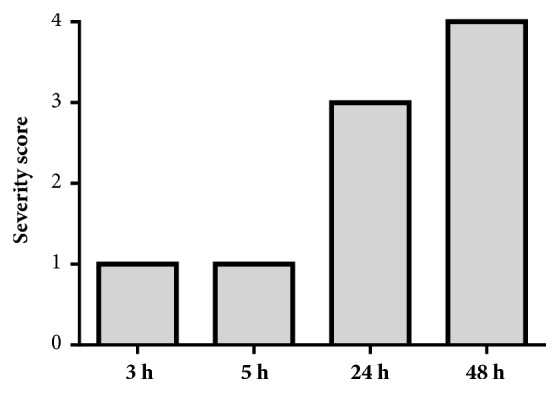
Adult* S. mansoni* severity scores after exposure to 1.25 *μ*g/mL of the acetone fraction derived from* E. ivorense* methanol leaf extracts.

Upon completion of the somule screens, extracts and fractions that exhibited phenotypic changes at the lower concentrations (from 5 to 0.3126 *μ*g/mL) were selected and screened at 1.25 *μ*g/mL against adults (Figure 4).

**Figure 5:**
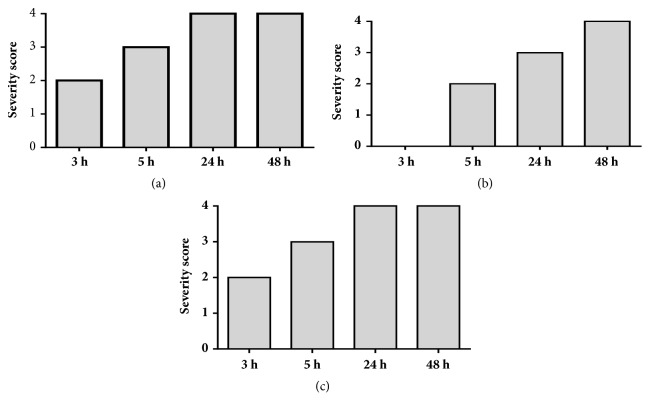
Adult* S. mansoni* severity scores after exposure to 1.25 *μ*g/mL of fractions derived from the methanol bark extract of* E. ivorense*. (a) 50% methanol fraction, (b) methanol bark extract, and (c) acetone fraction.

**Figure 6:**
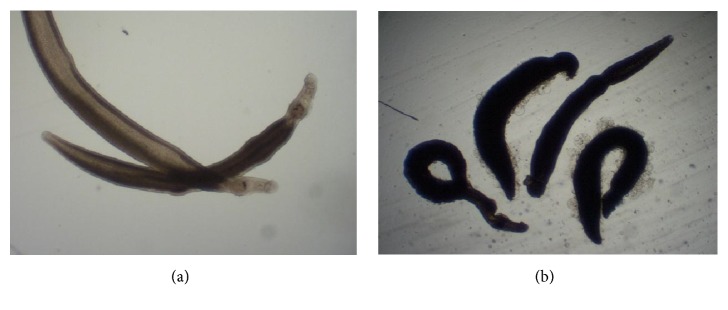
Examples of adult* S. mansoni* males after exposure to 1.25 *μ*g/mL of* E. ivorense* methanol bark extract fractions. (a) Control worms (each worm is approximately 500 to 700 *μ*m long); (b) damaged worms in the presence of the 50% v/v methanol fraction after 24 h. Note the marked darkening and the damage (blebbing) to the tegumental surface.

The acetone fraction of the methanol bark extract of* E. ivorense* caused the adult worms to become uncoordinated by 3 and 5 h, giving a score of 1. After 24 h, the worms remained uncoordinated and, in addition, had become dark with an inability to adhere to the floor of the well, yielding a score of 3. By the end of 48 h incubation period, the worms were dead and exhibited tegumental (surface) damage (a score of 4) (Figures 5 and 6).

Turning to the methanol bark extracts, it was observed after 3 h that in the presence of the 50% v/v methanol fraction, the worms had become uncoordinated with an inability to adhere to the base of the well (yielding a score of 2). By 5 h, the worms had become slightly shrunk in addition to the previous effects, thus increasing the score to 3. By 24 h, the worms exhibited tegumental damage and markedly slowed motility (score of 4). After 48 h, all of the worms were dead. The methanol bark extract caused no effects after 3 h. At 5 h, however, the worms were uncoordinated and not adhering to the well floor (a score of 2). At 24 h, the worms were damaged in tegumental manner and by 48 h were dead (in each case a score of 4). Finally, in the presence of the acetone fraction after 3 h, adult worms were uncoordinated and not adhering to the well floor, yielding a score of 2. By 5 h, the worms had also shrunk (a score of 3). After 24 h, the worms were dead with tegumental damage (score of 4) (Figure 6).

## 4. Discussion

For* S. mansoni* somules, the phenotypic effects, and corresponding severity scores, were generally observed to be dose-dependent. de Moraes et al. [25] and Long et al. [17] similarly reported dose-dependent activity against* S. mansoni *for piplartine and polo-like kinase inhibitors, respectively. It was also observed that, for both developmental stages, the phenotypic effects were dynamic and time-dependent (severity scores can increase or decrease or the qualitative nature of the effect may change), although, in general, the longer the time of contact, the more pronounced the effects and the higher the severity score. For example, this was observed for somules with the acetone fraction of methanol bark extract of* E. ivorense*. At a concentration of 0.625 *μ*g/mL after 24 h, the severity score was 2, but after 48 h the score had increased to 4. Similar observations were made with the ethyl acetate fractions and acetone fraction of the methanol leaf extract of the same plant.

The current antischistosomal drug, praziquantel, causes alterations to the tegumental surface of adult worms which makes the parasite vulnerable to host immune killing [26–28]. Methanol fractions and extract of* E. ivorense *bark and leaf also caused tegumental damage which is encouraging in light of the need for new antischistosomal agents, especially as many of the fractions and extracts also exhibited activity against somules, against which praziquantel is less active* in vivo *[24]. Any new drug molecule of interest should be active against all developmental stages of the parasite [7].

Secondary metabolites such as alkaloids, tannins, flavonoids, saponins, and sterols are responsible for the antimicrobial, analgesic, antiinflammatory, and antioxidant properties of medicinal plants [29, 30]. The activity exhibited by the algae extracts against the parasites could be as a result of the phytochemical constituents present in them. Our screening data suggest that the acetone fractions of both the bark and leaf exhibited the stronger activity. Secondary metabolites such as alkaloids, tannins, terpenoids, and flavonoids are found in* E. ivorense *as reported by Adu-Amoah et al. [14] and the activities observed may be attributable to these constituents. According to Molgaard et al. [31], extracts of* Abrus precatorius,* which exhibited significant activity against juvenile worms of* S. mansoni, *contain these phytochemical constituents. It is, therefore, possible that these secondary metabolites present in these extracts are responsible for the observed activity: the metabolites could be working synergistically or eliciting individual effects. Our future studies will include the bioactivity-guided isolation and characterization of the discrete compounds responsible for the current antischistosomal activities.

Finally, Adu-Amoah et al. [14] have reported that methanol leaf and bark extracts of* E. ivorense* (100, 300, and 1000 mg/kg) show no toxicity* in vitro* against HaCaT keratinocytes and* in vivo* in studies in male Wistar rats. Hence it is conceivable that the 1.25–100 *μ*g/mL concentrations that elicited activity in both parasite stages will not induce significant toxicity.

## 5. Conclusion

Methanol leaf and stem bark extracts and fractions of* E. ivorense *exhibited activity against both* S. mansoni* somules and adults. Of all the solvents used for the extraction and fractionation, the methanol extract of the stem bark, acetone fractions of the leaf and stem bark extracts, and the 50% methanol fraction of the stem bark extract showed the greatest activities against both stages. Considering the schistosomicidal effects against somules and adults of* S. mansoni*, the medicinal plant* E. ivorense* may offer a step forward in the search for novel antischistosomal agents, due to the urgent need for new drugs.

## Figures and Tables

**Table 1:** Descriptors and corresponding severity scores for the effect of extracts and fractions of *E. ivorense* on *S. mansoni*. Unless otherwise indicated, the descriptors employed are common to both somules and adults.

**Descriptor**	**Severity score**
Round	1
On sides (adults: loss of ability to adhere to the well surface)	1
Uncoordinated (adults)	1
Shrunk (adults)	1
Dark	1
Slow	1
Overactive	1
Immobile	1
Partial death (% somules dead /well)	1
Degenerate	4
Dead	4
Tegumental damage (adults)	4

Severity score: 1 (lowest) to 4 (highest).
